# Objective and perceived neighbourhood walkability: population-average associations with transportation and recreational physical activity in urban-dwelling Canadian adults

**DOI:** 10.1186/s12889-026-27408-y

**Published:** 2026-04-17

**Authors:** Levi Frehlich, Justin J. Lang, Stephanie A. Prince, Gavin R. McCormack

**Affiliations:** 1 Department of Community Health Sciences, Cumming School of Medicine, University of Calgary, Calgary, Canada; 2 Department of Family Medicine, Cumming School of Medicine, University of Calgary, Calgary, Canada; 3 Centre for Surveillance and Applied Research, Public Health Agency of Canada, Ottawa, Canada; 4 Alliance for Research in Exercise, Nutrition and Activity (ARENA), University of South Australia, Adelaide, South Australia Australia; 5 School of Epidemiology and Public Health, Faculty of Medicine, University of Ottawa, Ottawa, Canada; 6 O’Brien Institute for Public Health, University of Calgary, Calgary, Canada; 7 Libin Cardiovascular Institute, University of Calgary, Calgary, Canada; 8 Alberta Children’s Hospital Research Institute, University of Calgary, Calgary, Canada

**Keywords:** Adult, Environmental Design, Movement, Recreation, Transportation

## Abstract

**Background:**

The extent to which objective and perceived neighbourhood walkability together shape physical activity is not well understood. Using data from a national sample of urban-dwelling adults in Canada, our study examined whether perceived walkability moderated associations between objective walkability and physical activity undertaken for transportation (TPA) and recreation (RPA).

**Methods:**

National data from 3,995 adults (Canadian Health Measures Survey; 2016-2019) including self-reported sociodemographic characteristics, perceived walkability, and TPA and RPA were linked to objective walkability scores from the 2016 Canadian Active Living Environments (Can-ALE) dataset. Pooled and sex-stratified covariate-adjusted Hurdle models estimated associations between objective and perceived walkability (main and interaction effects), used to determine population-average weekly minutes of TPA and RPA.

**Results:**

Objective walkability was positively associated with TPA in the pooled (β = 9.39; 95% CI: 0.61 to 11.18) and sex-stratified samples (male β = 7.75; 95% CI: 5.42 to 10.08 and female β = 11.60; 95% CI: 8.88 to 14.31). Perceived walkability was positively associated with TPA in the pooled (β = 5.53; 95% CI:1.55 to 9.51) and male-only sample (β = 6.00; 95% CI: 0.31 to 11.68). Among males, perceived walkability was also associated with RPA (β = 8.13; 95% CI: 0.61 to 15.66). For males, the objective-by-perceived walkability interaction had a synergistic effect on TPA but an antagonistic effect on RPA. No interactions were observed among females.

**Conclusions:**

Objective and perceived walkability were positively associated with TPA. Perceived walkability was also linked to RPA among males. Their combined influence enhanced TPA but reduced RPA in males, suggesting physical and perceptual aspects of the neighbourhood built environment may interact differently across physical activity domains and sex.

**Supplementary Information:**

The online version contains supplementary material available at 10.1186/s12889-026-27408-y.

## Background

Many adults globally accumulate less physical activity than recommended for optimal health [1]. In Canada, only about half of adults achieve the 150 min of moderate-to-vigorous intensity physical activity (MVPA) per week, recommended by the Canadian 24-hour Movement Guidelines [2]. Given its broad health benefits and protective effects against chronic disease [3], identifying population-level strategies that promote physical activity remains essential. The built environment is a determinant of health [4], shaping the health behaviours of individuals and populations, including physical activity [5]. The majority of an adult’s daily life occurs near home, thus residential neighbourhoods are an important setting for promoting physical activity [6]. Accumulated evidence over the last 2–3 decades demonstrates consistent associations between physical activity and built characteristics, including neighbourhood walkability [7].

Neighbourhood walkability reflects a combination of built environment characteristics that support physical activity, and in particular walking and cycling [7, 8]. Walkability can be assessed via subjective (i.e., self-reported perceptions) and objective (i.e., street audits, spatial and geographical information systems data) [9] evaluations of the built environment. Both perceived and objective neighbourhood walkability are associated with physical activity, though findings suggest stronger and more consistent associations with transportation versus recreational physical activity [10, 11], which can contribute to meeting recommended physical activity levels [12]. Despite this evidence and their low-to-moderate correspondence [10, 11], measures of perceived and objective walkability are not interchangeable. Studies have observed a notable degree of mismatch between how individuals perceive the walkability of their neighbourhood and its objective walkability [13, 14]. Studies examining both concurrently, have reported independent associations with physical activity [10, 11, 15], while other findings suggest perceived walkability may mediate associations between objective walkability and physical activity [16, 17]. Few studies have investigated potential interaction effects between perceived and objective walkability, and findings among those that have are generally mixed [13, 15, 17–19].

Perceived walkability has the potential to amplify or attenuate the effects of objective walkability on transportation (TPA) and recreational (RPA) physical activity. For instance, Desgeorges et al. [15] reported that objectively assessed walkability and facility access predicted the duration, but not participation, of active commuting and for errands only among adults perceiving high walkability and facility access. In their prospective study, Gebel et al. [13] similarly found that adults living in objectively high-walkable areas who perceived low walkability showed greater declines in transportation walking, whereas mismatch between perceived and objective walkability was unrelated to leisure walking [13]. Jack and McCormack [18] found that higher perceived utilitarian destination mix strengthened the relationship between objective walkability and participation in neighbourhood transportation walking, with additional interactions observed between perceived infrastructure, safety, and duration of transportation walking [18]. Bracy et al. [19] identified positive interactions between objective walkability, nearby parks, and perceived pedestrian safety, and negative interactions with perceived crime safety for accelerometer-measured activity. Cerin et al. [17] found that objective intersection density, land-use mix, and retail access associated with accelerometer-measured MVPA only when perceived pedestrian, traffic, and crime safety were high. Among these studies, few included dimension-specific measures of physical activity (i.e., participation and duration) [15, 18], examined different physical activity domains (i.e., transportation and recreation) [13, 15, 18], or tested interactions including perceived and objective walkability [13, 15, 19].

Physical activity is a multidimensional behaviour characterized by several key dimensions, including participation, duration, and intensity that may each respond differently to neighbourhood walkability [15, 18, 20]. To date, studies examining associations between neighbourhood walkability and other related built characteristics and physical activity have typically modelled these dimensions separately, often focusing on participation as a dichotomous outcome (e.g., any versus none) and duration (e.g., minutes per day) as a continuous outcome [8, 15, 18, 21]. Although this approach can address the issue of excess zeros and positive skew common in self-reported physical activity data [22], it assumes independence between participation and duration, limiting interpretation of the overall influence of walkability on physical activity. Because participation precedes duration and both may be shaped by the built environment, estimating total (i.e., population-average) effects is important from a public health perspective. Furthermore, although engaging in any physical activity confers health benefits, public health guidelines emphasize accumulated weekly duration (e.g., ≥ 150 min per week).

Two-equation approaches (e.g., Heckman sample-selection model, Cragg two-part Hurdle model) can jointly represent physical activity behaviour as sequential processes (i.e., equations for participation and duration) [23–26]. McCormack et al. [24] used a Heckman model to examine associations between sidewalk length and minutes of TPA and RPA conditional on participation. Other studies have similarly applied the Heckman framework to investigate correlates and account for selection processes in physical activity outcomes [27]. Heckman models are suited to scenarios where zeros arise because outcomes are unobserved (or latent) and a variable influences the decision to participate but not the amount of physical activity once participating. In contrast, Hurdle models estimate the two equations separately, typically assuming independent errors, and obtain unconditional, population-average effects by combining the two parts; the same unconditional decomposition can also be computed under Heckman. The two-part Hurdle model treats zeros as genuine (observed) non-participation, estimates participation and duration separately, and combines them to yield population-average effects with fewer assumptions, translating model coefficients into interpretable measures that describe how correlates influence both the probability of participation and the expected physical activity duration among those who participate. In essence, the Hurdle model merges the estimate for participation and duration of physical activity, without dropping those who did not participate in physical activity, to give an overall estimate of association. Hurdle models have been used in studies investigating sociodemographic correlates of physical activity [26, 28, 29], how individual domains of perceived built characteristics are associated with physical activity [30], as well as how dense versus large metropolitan areas are associated with time spent in different activities outside of home [31]. To our knowledge, Hurdle models have not been used in the context of estimating population-average effects of walkability on physical activity.

The aim of our study was to examine whether perceived walkability moderated associations between objective walkability and TPA and RPA among urban-dwelling adults in Canada. Given evidence of potential sex differences in built environment-physical activity associations [21], we examined these associations for all participants, as well as males and females, separately. Our hypothesis was that higher perceived neighbourhood walkability will result in synergistic positive associations between higher objectively measured walkability and physical activity, with stronger associations present for TPA compared to RPA.

## Methods

### Data sources

#### Canadian Health Measures Survey

Our study involved a secondary analysis of cross-sectional data from the Canadian Health Measures Survey (CHMS) (Cycle 5: 2016–2017 and Cycle 6: 2018–2019) linked to walkability and area-level deprivation data from the Canadian Urban Environmental Health Research Consortium (CANUE). A detailed description of the CHMS sample design and data collection procedures is presented elsewhere [32–36]. Briefly, the CHMS is a nationally representative survey of individuals aged 3–79 years in all 10 provinces using a three-staged sample design (i.e., collection site, dwelling, and individual) [32–36]. The CHMS includes household interviews that capture sociodemographic information, self-reported physical activity, and neighbourhood perceptions, among other health data. Response rates for Cycles 5 and 6 household interviews were 90% (*n* = 7,944) and 89% (*n* = 8,286), respectively. We excluded those with postal codes mapped to rural residents as the relationship between the built environment and physical activity in Canada differ between rural and urban adults [37, 38]. Our analysis included complete case data from urban Canadian adults ($\:\ge\:$18 years) who reported being able to walk unassisted and who were not pregnant. Health Canada and the Public Health Agency of Canada’s Research Ethics Board approved the CHMS [32–36].

### Variables

#### Objective neighbourhood walkability

Objective neighbourhood walkability was assessed using the Can-ALE [39]. The Can-ALE dataset includes three built environment variables: street connectivity (number of ≥ 3-way intersections/km^2^), residential density (number of dwellings/km^2^), and destination density (number of points of interest) estimated within a 1-kilometer circular buffer around the centroid of each dissemination area including urban and rural residence. A dissemination area includes a population of approximately 400–700 individuals and is the smallest standard geographical unit used by Statistics Canada [40]. The Can-ALE index - an aggregate measure representing neighbourhood walkability - was derived by standardizing each of the three variables nationally and summing them into a single score. To obtain a measure of variability in walkability for our sample of urban adults, we applied the same approach but standardized the variables using sample-specific z-scores (mean centered to 0 with a standard deviation of 1), to derive a study-specific walkability index. The Can-ALE index is positively associated with physical activity for different purposes (i.e., work, school, shopping, walking, and recreation) [41–43] as well as physical activity-related outcomes, including components of health-related fitness [44–46].

#### Perceived neighbourhood walkability

Perceived neighbourhood walkability was assessed using items from the Physical Activity Neighbourhood Environment Scale (PANES) [47]. The full-version of the PANES scale has 17 items including 7 core, 4 recommended, and 6 optional questions that capture perceptions of different built attributes related to walkability in the neighbourhood (i.e., defined as 10–15 min walk from home) [47]. CHMS Cycles 5 and 6 featured a Neighbourhood Built Environment module that included 7 core and 4 non-core PANES items. We used 6 of the 7 core items that make up the PANES Built Environment Index (PANES-BEI), an overall measure of perceived neighbourhood walkability [47]. The 6 core items captured perceptions of housing types, retail destinations, transit stops, presence of sidewalks, facilities to bicycle to, and low cost or free recreational facilities. All items included a 4-point Likert response from strongly disagree to strongly agree, with the exception of the question on housing type (response options included: ‘detached single-family housing’, ‘townhouses, row houses, apartments, or condos of 2–3 stories’, ‘mix of single-family residences and townhouses, row houses, apartments or condos’, ‘apartments or condos of 4–12 stories’, ‘apartments or condos of more than 12 stories,’ and ‘don’t know/not sure’). In alignment with the PANES scoring protocol [47] the 6 core items were dichotomized as ‘0’ or ‘1’(housing type was dichotomized as 0 for detached single-family housing, and 1 for any other response) representing disagreement or agreement, respectively, and summed to derive the PANES-BEI. The PANES-BEI ranges from 0–6 with higher PANES-BEI scores reflecting higher perceived walkability. Internal consistency of the PANES-BEI in our sample was acceptable (Cronbach’s α: total sample = 0.60; males = 0.57; females = 0.62) and congruent with estimates reported elsewhere [48, 49]. Among adults, associations have been observed between the PANES-BEI and physical activity [48, 50, 51].

#### Physical activity

Self-reported weekly TPA was assessed by asking participants “*In the last seven days*,* that is from last this day last week to yesterday*,* did you use active ways like walking or cycling to get to places such as work*,* school*,* the bus stop*,* the shopping centre or to visit friends?*” Similarly, self-reported RPA was assessed by asking participants “*In the last seven days*,* did you do sports*,* fitness or recreational physical activities*,* organized or non-organized*,* that lasted a minimum of 10 continuous minutes? Examples are walking*,* home or gym exercise*,* swimming*,* cycling*,* running*,* skiing*,* dancing and all team sports*.” [52]. For those reporting TPA and RPA participation, daily minutes of these activities was captured and each summed to create estimated TPA and RPA weekly duration variables.

#### Covariates

Our analysis included several covariates from the CHMS data known to associate with the outcome: sex (male or female), age (years), university education (yes or no), landed immigrant (yes or no), currently working at a job (yes or no), children living in the household under 15 years of age (yes or no), marital status (married/common-law, widowed/separated/divorced, or single), access to a motor vehicle (yes or no), and ethnicity (white or non-white). The Material and Social Deprivation Index (MSDI) at the postal code was also included as a covariate. MSDI includes census-derived dissemination area-level material deprivation (i.e., income, education, and employment) and social deprivation (i.e., proportions of household that report being single-parent families or those living alone, those separated, and individuals divorced, or widowed) [53, 54].

### Statistical analysis

All analyses were conducted with the pooled, as well as sex-specific samples. Survey weights were provided by Statistics Canada. For descriptive statistics, we used weighted sample data to estimate means for continuous variables or proportions for categorical variables. We also applied bootstrap weights (500 replicates) to account for the complex sampling design when calculating the 95% confidence intervals (CI), for sociodemographic characteristics, objective and perceived walkability, and TPA and RPA participation and duration outcomes. Using unweighted sample data, we undertook exponential Cragg Hurdle models, which included a probit model for estimating participation and log-linear model for estimating duration, to derive the unconditional (population-average) marginal main and interaction effects of objective and perceived walkability on TPA and RPA.

To assess effect modification, multiplicative objective-by-perceived walkability interaction terms were included as predictors in the probit and log-linear components of the Hurdle model. Pairwise contrasts compared differences in the slopes of the associations between objective walkability and physical activity across the lowest (PANES-BEI = 0), mid-point (PANES-BEI = 3), and highest (PANES-BEI = 6) perceived walkability levels. To evaluate the sensitivity of results to model specification, additional models were estimated in which the interaction term was excluded from either the participation (probit) or duration (log-linear) component. A less conservative significance threshold (*p*<0.10) was used to assess interaction effects, whereas the conventional threshold (*p*<0.05) was applied for main effects. Less conservative p-value thresholds have been used elsewhere when examining interactions between the built environment, sociodemographic characteristics, and physical activity [55–58]. Although point estimates from the probit and log-linear parts of the Hurdle model provide useful insights into the direction of association, they are not directly interpretable; instead, they were used post-estimation to derive unconditional (population-average) marginal effects of perceived and objective walkability on TPA and RPA. Models were fully-adjusted for covariates with simultaneous adjustment for objective and perceived walkability, and for all coefficients, 95% confidence intervals were estimated using robust standard errors (Huber-White sandwich estimators). Urban area was based on household postal code. Point estimates and confidence intervals for participants who were excluded based on missing data were explored. Analyses were conducted using Stata version 18 (StataCorp LLC, College Station, TX, USA).

## Results

### Sample characteristics

Table 1 provides the weighted total and sex-stratified sample characteristics. The analysis included an unweighted sample of 3,995 adults (males *n* = 2,081, females *n* = 1,914). The pooled sample had a mean age of approximately 47 years which was similar among males and females. A higher proportion of females than males reported having a university education. Nearly all participants, regardless of sex, had access to a motor vehicle. The pooled sample had a mean Can-ALE index (objective walkability) score of 0.28, with corresponding means of 0.25 for males and 0.30 for females. The estimated mean PANES-BEI (perceived walkability) score was 4.53 in the pooled sample, and 4.52 for males, and 4.53 for females. The Pearson’s correlations between objective and perceived walkability scores were moderate (pooled sample: *r* = 0.41; males: *r* = 0.41; females: *r* = 0.43). Among those participating in TPA the mean was 181.6 min/week, with males averaging 183.4 min/week and females 180.0 min/week. Moreover, those participating in RPA reported an average of 214.4 min/week, with males averaging 240.1 min/week and females 187.8 min/week. Compared to excluded cases, the included sample reported lower perceived walkability, was older, and had a higher proportion of individuals with a university education, currently working, and married (Table S1).

**Table 1:** Weighted sample characteristics

Characteristic	Units	Total	Males	Females
		Estimate (95% CI)	Estimate (95% CI)	Estimate (95% CI)
Neighbourhood built environment				
Objective walkability (Can-ALE)	Standardized ^a^	0.28 (-0.58 to 1.13)	0.25 (-0.64 to 1.14)	0.30 (-0.52 to 1.13)
Perceived walkability (PANES-BEI)	Summed index ^b^	4.53 (4.33 to 4.72)	4.52 (4.29 to 4.75)	4.53 (4.32 to 4.74)
Material deprivation index	Standardized ^a^	-0.02 (-0.02 to -0.01)	-0.01 (-0.02 to -0.01)	-0.02 (-0.03 to -0.01)
Social deprivation index	Standardized ^a^	0.00 (-0.00 to 0.01)	0.00 (-0.00 to 0.01)	0.00 (-0.00 to 0.01)
Physical activity				
Transport-related - participated	%	44 (38 to 49)	41 (35 to 47)	46 (40 to 53)
Transport-related duration	min/week	181.6 (161.5 to 201.8)	183.4 (150.3 to 216.5)	180.0 (160.4 to 199.6)
Recreational - participated	%	50 (46 to 54)	50 (45 to 55)	50 (45 to 56)
Recreational - duration	min/week	214.4 (194.8 to 234.0)	240.1 (215.5 to 264.6)*	187.8 (166.1 to 209.6)*
Sociodemographic characteristics				
Age	years	47.0 (46.3 to 47.6)	46.8 (45.9 to 47.6)	47.2 (46.3 to 48.1)
University education	%	75 (71 to 78)	70 (65 to 74)*	80 (76 to 83)*
Marital status				
Married	%	55 (50 to 59)	56 (51 to 61)	53 (49 to 57)
Widowed/separated/divorced	%	22 (19 to 25)	20 (16 to 23)	24 (20 to 28)
Single	%	24 (21 to 27)	24 (21 to 28)	23 (20 to 27)
Any children under 15 in house	%	34 (31 to 36)	34 (30 to 38)	34 (31 to 37)
Currently working at a job	%	67 (65 to 69)	72 (69 to 74)*	62 (58 to 66)*
Any access to a motor vehicle	%	93 (91 to 95)	95 (92 to 97)	92 (89 to 94)
Landed immigrant	%	36 (28 to 45)	37 (28 to 48)	34 (26 to 43)
Ethnicity - White	%	72 (63 to 79)	69 (60 to 78)	75 (66 to 82)

Statistics Canada survey weights and bootstrap replicates were used Continuous variables reported as mean and 95% confidence interval, categorical variables reported as percent and 95% confidence intervalWeighted/unweighted sample: total *n* = 16,564,369/3,995; male *n* = 8,42,211/2,081; female *n* = 8,122,157/1,914

Can-ALE: Canadian Active Living Environments Index, PANES-BEI: Physical Activity Neighbourhood Environment Scale - Built Environment Index.

^a^ Standardized scores (z scores)

^b^ Six questions dichotomised and summed for a range of 0–6

*Bolded values indicate no confidence interval overlap between males and females

### Objective and perceived walkability independent main effects

Table 2 shows the estimated associations between objective and perceived walkability and self-reported weekly TPA and RPA from the Hurdle models. In the pooled sample, objective walkability was positively associated (*p*<0.05) with both participation in and weekly minutes of TPA, corresponding to a population-average marginal increase of 9.39 min/week (95% CI: 0.61 to 11.18) per one-unit increase in objective walkability. In the same model, perceived neighbourhood walkability was positively associated (*p*<0.05) with participation but not weekly minutes of TPA, corresponding to a population-average marginal increase of 5.53 min/week (95% CI: 1.55 to 9.51) per one-unit increase in perceived walkability. In the pooled sample, there were no significant associations between either objective or perceived walkability with RPA participation or duration.

**Table 2:** Hurdle model estimated associations between objective walkability, perceived walkability, and self-reported weekly physical activity (main effects)

	Physical activity domain	Walkability measure	Participation	Duration	Margin^
			Probit coefficient (95% CI)	Log-linear coefficient (95% CI)	Linear coefficient (95% CI)
All adults (*n* = 3995)	Transportation	Perceived	0.08 (0.05 to 0.12)*	0.01 (-0.04 to 0.06)	5.53 (1.55 to 9.51)*
		Objective	0.10 (0.08 to 0.13)*	0.05 (0.03 to 0.06)*	9.39 (7.61 to 11.18)*
	Recreation	Perceived	-0.00 (-0.034 to 0.03)	0.03 (-0.00 to 0.06)	3.25 (-1.50 to 8.00)
		Objective	0.01 (-0.01 to 0.03)	-0.01 (-0.02 to 0.01)	0.05 (-2.33 to 2.44)
Males (*n* = 2081)	Transportation	Perceived	0.11 (0.06 to 0.16)*	-0.01 (-0.07 to 0.06)	6.00 (0.31 to 11.68)*
		Objective	0.07 (0.05 to 0.10)*	0.05 (0.02 to 0.07)*	7.75 (5.42 to 10.08)*
	Recreation	Perceived	0.01 (-0.03 to 0.06)	0.05 (0.00 to 0.10)*	8.13 (0.61 to 15.66)*
		Objective	0.01 (-0.02 to 0.03)	-0.00 (-0.02 to 0.02)	0.49 (-3.19 to 4.18)
Females (*n* = 1914)	Transportation	Perceived	0.05 (0.00 to 0.10)*	0.02 (-0.04 to 0.09)	4.87 (-0.73 to 10.47)
		Objective	0.15 (0.11 to 0.18)*	0.04 (0.02 to 0.07)*	11.60 (8.88 to 14.31)*
	Recreation	Perceived	-0.02 (-0.07 to 0.03)	0.01 (-0.04 to 0.05)	-0.72 (-6.55 to 5.11)
		Objective	0.01 (-0.01 to 0.04)	-0.01 (-0.03 to 0.01)	-0.31 (-3.31 to 2.69)

^^^ Unconditional (population-average) marginal effect representing the total effect of the walkability measure on expected physical activity minutes across the entire sample, incorporating its effects on the probability of participation and duration among participants adjusting for all covariates

Covariates: age, material and social deprivation indices, education, immigration status, working status, children in the household, marital status, access to a motor vehicle, and ethnicity, with simultaneous adjustment for objective and perceived walkability. Covariates adjusted for both participation and duration

*CI* Confidence Interval

*Bolded values indicate *p* < 0.05

For the sex-stratified analysis, objective walkability was positively associated (*p*<0.05) with both participation and weekly minutes of TPA, corresponding to a population-average marginal increase per one-unit increase in objective walkability of 7.75 min/week (95% CI: 5.42 to 10.08) for males and 11.60 min/week (95% CI: 8.88 to 14.31) for females. Sex-stratified analysis also revealed positive associations (*p*<0.05) between perceived neighbourhood walkability and TPA participation but not duration; however, a significant population-average marginal increase per one-unit increase in perceived walkability was only observed for males (β = 6.00 min/week; 95% CI: 0.31 to 11.68). There were no associations between either objective or perceived walkability with weekly minutes of RPA for females; however, perceived walkability was positively associated (*p*<0.05) with RPA duration among males, corresponding to a population-average marginal increase of 8.13 min/week (95% CI: 0.61 to 15.66) per one-unit increase in perceived walkability (Table 2).

### Objective-by-perceived walkability interaction effects

Tables 3,4, and 5 present the pairwise contrasts quantifying differences in the slope of the association between objective walkability and weekly minutes of RPA and TPA across low (PANES-BEI = 0), moderate (PANES-BEI = 3), and high (PANES-BEI = 6) perceived walkability. Table 3 shows results estimated from models that included the objective-by-perceived walkability interaction term in both the participation and duration components. There was evidence of interaction (*p*<0.10) for the association between objective walkability and minutes of RPA, such that the association was weaker in magnitude among adults perceiving their neighbourhood as highly walkable relative to those perceiving it as moderately walkable, in the pooled sample (β = -6.47; 95% CI: -13.65 to 0.71, *p* = 0.08) and among males (β = -10.10; 95% CI: -21.49 to 1.29, *p* = 0.08). In addition, the association between objective walkability and minutes of TPA was stronger in magnitude among males that perceived their neighbourhood as moderately walkable compared with those perceiving it as low walkable (β = 2.97; 95% CI: -0.48 to 6.42, *p* = 0.09).

**Table 3:** Pairwise contrasts of population-average marginal effects^#^ of objective walkability on weekly physical activity by levels of perceived walkability, from models including objective-by-perceived walkability interactions^^^ (participation and duration components)

	Physical activity domain	Perceived walkability: Mod vs. Low	Perceived walkability: High vs. Low	Perceived walkability: High vs. Mod
		Linear coefficient (95% CI)	Linear coefficient (95% CI)	Linear coefficient (95% CI)
All adults (*n* = 3995)	Transportation	2.45 (-0.51 to 5.41)	5.48 (-2.51 to 13.48)	3.03 (-2.06 to 8.13)
	Recreation	-6.65 (-15.82 to 2.52)	-13.12 (-29.41 to 3.17)	-6.47 (-13.65 to 0.71)*
Males (*n* = 2081)	Transportation	2.97 (-0.48 to 6.42)*	6.59 (-3.38 to 16.55)	3.62 (-3.13 to 10.37)
	Recreation	-9.68 (-25.71 to 6.34)	-19.78 (-47.01 to 7.46)	-10.10 (-21.49 to 1.29)*
Females (*n* = 1914)	Transportation	2.18 (-2.54 to 6.90)	4.99 (-7.41 to 17.40)	2.81 (-4.91 to 10.53)
	Recreation	-3.62 (-14.08 to 6.83)	-6.83 (-25.80 to 12.14)	-3.20 (-11.75 to 5.34)

^#^Unconditional (population-average) marginal effect representing the total effect of the walkability measure on expected physical activity minutes across the entire sample, incorporating its effects on the probability of participation and duration among participants adjusting for all covariates

^^^ Interaction includes a multiplicative interaction term between objective and perceived walkability in both the participation and duration model

Covariates: age, material and social deprivation indices, education, immigration status, working status, children in the household, marital status, access to a motor vehicle, and ethnicity, with simultaneous adjustment for objective and perceived walkability. Covariates adjusted for both participation and duration

*PA* Physical Activity, *CI* Confidence Interval

Perceived walkability ranged from 0–6 (low = 0, mid-point = 3, high = 6)

*Bolded values indicate *p* < 0.10

**Table 4:** Pairwise contrasts of population-average marginal effects^#^ of objective walkability on weekly physical activity by levels of perceived walkability, from models including objective-by-perceived walkability interactions^^^ (duration component only)

	Physical activity domain	Perceived walkability: Mod vs. Low	Perceived walkability: High vs. Low	Perceived walkability: High vs. Mod
		Linear coefficient (95% CI)	Linear coefficient (95% CI)	Linear coefficient (95% CI)
All adults (*n* = 3995)	Transportation	2.11 (0.21 to 4.01)**	4.69 (-1.16 to 10.54)	2.58 (-1.39 to 6.54)
	Recreation	-5.05 (-12.07 to 1.98)	-10.10 (-22.77 to 2.57)	-5.05 (-10.74 to 0.64)*
Males (*n* = 2081)	Transportation	1.86 (-0.58 to 4.29)	3.95 (-3.92 to 11.82)	2.09 (-3.36 to 7.55)
	Recreation	-6.70 (-17.95 to 4.55)	-14.24 (-34.13 to 5.65)	-7.54 (-16.30 to 1.22)*
Females (*n* = 1914)	Transportation	2.49 (-0.62 to 5.59)	5.71 (-3.43 to 14.86)	3.23 (-2.82 to 9.27)
	Recreation	-3.14 (-11.99 to 5.70)	-5.92 (-21.82 to 9.98)	-2.77 (-9.86 to 4.31)

^#^Unconditional (population-average) marginal effect representing the total effect of the walkability measure on expected physical activity minutes across the entire sample, incorporating its effects on the probability of participation and duration among participants adjusting for all covariates

^^^ Interaction includes a multiplicative interaction term between objective and perceived walkability in the duration model

Covariates: age, material and social deprivation indices, education, immigration status, working status, children in the household, marital status, access to a motor vehicle, and ethnicity, with simultaneous adjustment for objective and perceived walkability. Covariates adjusted for both participation and duration

*PA* Physical Activity, *CI* Confidence Interval

Perceived walkability ranged from 0–6 (low = 0, mid-point = 3, high = 6)

*Bolded values indicate *p* < 0.10

**Bolded values indicate *p* < 0.05

**Table 5:** Pairwise contrasts of population-average marginal effects^#^ of objective walkability on weekly physical activity by levels of perceived walkability, from models including objective-by-perceived walkability interactions^^^ (participation component only)

	Physical activity domain	Perceived walkability: Mod vs. Low	Perceived walkability: High vs. Low	Perceived walkability: High vs. Mod
		Linear coefficient (95% CI)	Linear coefficient (95% CI)	Linear coefficient (95% CI)
All adults (*n* = 3995)	Transportation	1.76 (-1.05 to 4.58)	3.45 (-2.74 to 9.63)	1.68 (-1.70 to 5.06)
	Recreation	-1.09 (-4.16 to 1.99)	-2.39 (-9.19 to 4.41)	-1.31 (-5.04 to 2.42)
Males (*n* = 2081)	Transportation	2.65 (-1.02 to 6.32)	5.29 (-3.03 to 13.62)	2.65 (-2.07 to 7.36)
	Recreation	-1.57 (-5.48 to 2.34)	-3.80 (-13.22 to 5.62)	-2.22 (-7.76 to 3.31)
Females (*n* = 1914)	Transportation	1.09 (-2.92 to 5.10)	2.12 (-6.75 to 11.00)	1.03 (-3.84 to 5.90)
	Recreation	-0.33 (-4.88 to 4.22)	-0.69 (-10.11 to 8.72)	-0.36 (-5.23 to 4.51)

^#^Unconditional (population-average) marginal effect representing the total effect of the walkability measure on expected physical activity minutes across the entire sample, incorporating its effects on the probability of participation and duration among participants adjusting for all covariates

^^^ Interaction includes a multiplicative interaction term between objective and perceived walkability in the duration model

Covariates: age, material and social deprivation indices, education, immigration status, working status, children in the household, marital status, access to a motor vehicle, and ethnicity, with simultaneous adjustment for objective and perceived walkability. Covariates adjusted for both participation and duration.Perceived walkability ranged from 0–6 (low = 0, mid-point = 3, high = 6)

*PA* Physical Activity, *CI* Confidence Interval

Table 4 shows results from models that included the objective-by-perceived walkability interaction term in the duration component only. The association between objective walkability and weekly minutes of TPA was stronger in magnitude among adults perceiving their neighbourhood as moderately walkable compared with those perceiving it as low walkable (β = 2.11; 95% CI: 0.21 to 4.01, *p* = 0.03). In addition, the association between objective walkability and minutes of RPA was weaker in magnitude among those perceiving their neighbourhood as high walkable compared with those perceiving it as low walkable, among all adults (β = -5.05; 95% CI: -10.74 to 0.64, *p* = 0.08) and males only (β = -7.54; 95% CI: -16.30 to 1.22, *p* = 0.09). No indication of an objective-by-perceived walkability interaction effect was observed for models with the interaction term included in the participation component only (Table 5).

## Discussion

Our study aimed to estimate population-average effects and to determine whether perceived walkability moderated associations between objective walkability and TPA and RPA among urban-dwelling adults. Our analysis revealed important insights into the associations between neighbourhood walkability and domain-specific physical activity among adults in Canada. Our results suggest that objective and perceived walkability are positively associated with TPA, particularly among males. The evidence of interaction effects suggests that moderate perceived walkability may amplify, and high perceived walkability may attenuate, associations between objective walkability and physical activity. These interactions appeared to operate primarily through the duration of physical activity rather than through the probability of participation. Our findings reinforce previous evidence highlighting the importance of both objective and perceived neighbourhood walkability in shaping physical activity [7, 8, 10, 11]. Importantly, this study supports previous evidence suggesting that objective and perceived walkability may have different effects on the physical activity of adult males versus females [21]. Methodologically, using a Hurdle model to estimate population-average effects that jointly account for participation and duration as sequential but connected processes, our study provides estimates of the overall impact that changes in neighbourhood walkability (perceived and objective) may have on weekly minutes of TPA and RPA in adults.

Our results from models estimating main effects are consistent with previous research showing that both the objective and perceived neighbourhood built environment appear important for supporting physical activity [7, 8, 10, 11]. Similar to previous studies [7, 59], our findings suggest that the neighbourhood built environment may exert stronger influence on TPA than on RPA physical activity. In their synthesis of 116 systematic reviews of observational and experimental studies, Prince et al. [7] reported high-certainty evidence for positive associations between neighbourhood walkability and TPA. They also reported mixed associations between neighbourhood walkability and RPA, but that positive effects were more often observed when neighbourhood walkability was assessed via perceptions [7]. Our findings corroborate this pattern, showing that objective walkability was more consistently associated with TPA, whereas perceived walkability showed weaker and domain-specific associations. Objective walkability was positively associated with both participation in, and duration of, TPA among males and females, whereas perceived walkability was primarily related to participation in TPA and, among men, to the duration of RPA. Notably, in the pooled sample and for males only, objective and perceived walkability showed positive independent associations with population-average weekly minutes of TPA. Contrary to other Canadian studies examining RPA as an outcome (e.g., walking) [60, 61], perceived walkability was not associated with participation among males or females.

In our study, perceived walkability modified the associations between objective walkability and physical activity – specifically for duration of RPA in the pooled sample and among males, and for duration of TPA among males. Among females, perceived walkability did not significantly modify associations between objective walkability and TPA or RPA, though the direction of associations mirrored those for males. Like others, our finding suggest that the association between objective walkability and physical activity may partly depend on how individuals perceive their environment [15, 17–19]. Enhancing neighbourhood walkability, while beneficial for promoting physical activity, may have limited effects among individuals who continue to perceive their neighbourhood as low walkable. Consistent with previous studies [13, 15, 18], moderating effects of perceived walkability were more evident for TPA than RPA (significant effects for TPA at p<0.05 and weaker evidence for RPA at p<0.10), and that mismatches between perceived and objective walkability may negatively effect physical activity. This pattern may suggest a saturation effect, whereby enhancing walkability in initially unwalkable neighbourhoods substantially increases TPA, with diminishing returns once a moderate level of walkability is attained. Cerin et al.[62] examined data from 14 cities and ten countries, and found that population, intersection, and transit density has curvilinear relationships with TPA participation, providing evidence for this saturation hypothesis. To maximize walkability’s impact, efforts should pair physical improvements with strategies that foster positive perceptions of the built environment. Context-tailored interventions aimed at encouraging engagement in the outdoor environment and increasing neighbourhood awareness (e.g., walking maps) show promise for modifying environmental perceptions and physical activity [63–66].

Like others [15, 18], we found that interactions between objective and perceived walkability appear to operate primarily through associations with duration of, rather than participation in, physical activity. This raises an important consideration as it might suggest perceptions of walkability are a consequence of performing physical activity in the neighbourhood, rather than a cause of time spent physically active. Quantitative and qualitative evidence suggest that physical activity interventions that increase exposure to the neighbourhood built environment might lead to enhanced awareness and favourable perceptions of walkability characteristics [67–69]. The influence of physical activity on neighbourhood perceptions is plausible given that personal history, beliefs, attitudes, and contextual experiences (e.g., interaction with the built environment) shape how individuals perceive their surroundings [70]. More longitudinal and experimental studies are needed to unpack the temporal relationship between neighbourhood perceptions and physical activity.

### Strengths and limitations

Our study has several strengths including using established objective and perceived measures of walkability. Our analytical strategy allowed for the estimations of population-average effects of walkability on TPA and RPA, accounting for different relationships with participation and duration, while also allowing for testing for interactions between perceived and objective walkability. Although allowing for separate domains (transportation and recreation) and dimensions (participation and duration) to be examined, self-reported physical activity is vulnerable to memory errors and reporting bias [71]. Self-reported TPA captured both walking and cycling, thus did not provide an opportunity to investigate mode-specific physical activity and walkability relationships [72]. Moreover, our objective measure of walkability was constructed at a 1-kilometer circular buffer limiting our assessment of varying geographical sizes of environmental exposure, commonly known as the Modifiable Area Unit Problem [73]. Our restriction to urban adults, inclusion criteria, and complete case analysis limits generalizability of our findings. Our secondary analysis included cross-sectional data precluding causal inference due to the inability to establish temporal order, including the potential influence of residential self-selection on both main and interaction effects between objective walkability, perceived walkability, and physical activity.

## Conclusions

Our study contributes to the growing evidence that objective and perceived neighbourhood environment influence physical activity both independently and jointly. Perceptions may shape how objective walkability influences physical activity participation versus duration. Both males and females may benefit from supportive built environments through increases in TPA, with perceptions mattering more for males. Moreover, males may also benefit through more RPA if they perceive their neighbourhood as more walkable. Future research should assess objective and perceived built environment together to better capture their combined effects on physical activity. Understanding these interactions is important for ascertaining whether physical activity promotion strategies should target residents’ perceptions, changes to the physical built environment (i.e., urban design), or both.

## Supplementary Information

Supplementary Material 1. Table S1. Weighted sample characteristics between included and excluded respondents.

## Data Availability

Data used in this project were provided by Statistics Canada and accessed through one or more of the RDCs (Research Data Centres) in the CRDCN. Because of the confidential nature of these microdata, they cannot be shared. Researchers in Canada working at one of CRDCN’s member institutions can access the data at no additional cost to the researcher. Other researchers will have to pay cost-recovery to access the data. Access to the data is subject to a background check and research approval process. The protocols for data access, including fees for researchers at non-CRDCN institutions, can be found on the CRDCN website.

## Abbreviations

CANUE: Canadian Urban Environmental Health Research Consortium
Can-ALE: Canadian Active Living Environments
CHMS: Canadian Health Measures Survey
MSDI: Material and Social Deprivation Index
MVPA: Moderate-to-vigorous intensity physical activity
PANES: Physical Activity Neighbourhood Environment Scale
BEI-PANES: PANES Built Environment Index
RPA: Recreational physical activity
TPA: Transportation physical activity
