# Supplementary material for: Objective and perceived neighbourhood walkability: population-average associations with transportation and recreational physical activity in urban-dwelling Canadian adults
Source: BMC Public Health. 2026 Apr 17;26:1734. doi: 10.1186/s12889-026-27408-y (PMC13220403; doi:10.1186/s12889-026-27408-y)
Supplement: Supplementary file 1 — Supplementary Material 1. Table S1. Weighted sample characteristics between included and excluded respondents. [file 12889_2026_27408_MOESM1_ESM.docx]

**Supplementary Table**

|  |  |  |  |  |
| --- | --- | --- | --- | --- |

| **Table S1. Weighted sample characteristics between included and excluded respondents** | | | | | |  |
| --- | --- | --- | --- | --- | --- | --- |
|  | | |  | **Included^^^** | **Excluded^#^** | **Excluded** |
| **Characteristic** | | | **Units** | **Estimate (95% CI)** | **Estimate (95% CI)** | **Weighted/unweighted sample (n)** |
| Neighbourhood built environment | | |  |  |  |  |
|  | Objective walkability (Can-ALE) | | Standardized ^a^ | 0.28 (-0.58 to 1.13) | 0.69 (-0.55 to 1.92) | 6,545,785/1,433 |
|  | Perceived walkability (PANES-BEI) | | Summed index ^b^ | **4.53 (4.33 to 4.72)*** | **5.08 (4.88 to 5.27)*** | 4,491,007/918 |
|  | Material deprivation index | | Standardized ^a^ | -0.02 (-0.02 to -0.01) | 0.00 (-0.01 to 0.01) | 5,557,163/1,242 |
|  | Social deprivation index | | Standardized ^a^ | 0.00 (-0.00 to 0.01) | 0.02 (0.01 to 0.02) | 5,557,163/1,242 |
|  | | |  |  |  |  |
| Physical activity | | |  |  |  |  |
|  | Transport-related - participated | | % | 44 (38 to 49) | 49 (40 to 58) | 6,586,568/1,436 |
|  | | Transport-related duration | min/week | 181.6 (161.5 to 201.8) | 207.5 (176.4 to 238.5) | 3,384,655/762 |
|  | Recreational - participated | | % | 50 (46 to 54) | 44 (40 to 49) | 6,585,377/1,440 |
|  | | Recreational - duration | min/week | 214.4 (194.8 to 234.0) | 246.3 (179.5 to 313.2) | 2,924,621/623 |
|  | | |  |  |  |  |
| Sociodemographic characteristics | | | % |  |  |  |
|  | Age | | years | **47.0 (46.3 to 47.6)*** | **42.7 (41.5 to 44.0)*** | 6,623,375/1,452 |
|  | University education | | % | **75 (71 to 78)*** | **59 (53 to 65)*** | 6,401,497/1,412 |
|  | Marital status | |  |  |  | 6,592,166/1,450 |
|  | | Married | % | **55 (50 to 59)*** | **44 (40 to 49)*** |  |
|  | | Widowed/separated/divorced | % | 22 (19 to 25) | 22 (19 to 25) |  |
|  | | Single | % | **24 (21 to 27)*** | **34 (29 to 39)*** |  |
|  | Any children under 15 in house | | % | 34 (31 to 36) | 30 (25 to 35) | 6,623,375/1,452 |
|  | Currently working at a job | | % | **67 (65 to 69)*** | **55 (48 to 63)*** | 6,623,375/1,452 |
|  | Any access to a motor vehicle | | % | 93 (91 to 95) | 89 (80 to 94) | 3,919,101/864 |
|  | Landed immigrant | | % | 36 (28 to 45) | 33 (24 to 44) | 5,967,792/1,335 |
|  | Ethnicity - White | | % | 72 (63 to 79) | 65 (53 to 76) | 5,930,907/1,284 |
| Can-ALE: Canadian Active Living Environments Index, PANES-BEI: Physical Activity Neighbourhood Environment Scale - Built Environment Index. Statistics Canada survey weights and bootstrap replicates were used. Continuous variables reported as mean and 95% confidence interval, categorical variables reported as percent and 95% confidence interval.  Bolded values indicate no 95% confidence interval overlap.  ^^^Included was a complete case analysis n=16,564,369/3,995 based on no missing data  ^#^Excluded n varies by characteristic. Participants were excluded if one or more characteristics were missing.  ^a^ Standardized scores (z scores)  ^b^ Six questions dichotomised and summed for a range of 0-6.  *Bolded values indicate no confidence interval overlap between included and excluded respondents. | | | | | | |
